# Dr. John Davidson Hill

**Published:** 1892-04

**Authors:** 


### Obituary.

Dr. John Davidson Hill died at his residence in Buffalo on the 27th day of February, 1892, in the seventieth year of his age. The deceased had been for upwards of forty years a prominent practitioner of medicine in this city, and had attained a degree of eminence which placed him in the first rank. He had been President of the Medical Society of the County of Erie, was member of the board of managers of the Buffalo State Hospital, and had occupied many other places of honor in both business and professional capacities. He was one of the last representatives of the many prominent physicians of his period, and his loss will be long deeply felt by a large circle of friends and patrons. Under the head of "Society Proceedings" we publish in this issue the action of the Medical Society of the County of Erie relating to this melancholy event.

